# Natural Language Understanding to Assess Oral Health‐Related Quality of Life: A Cross‐Sectional Study Incorporating a Mixed Methods Approach

**DOI:** 10.1111/joor.13986

**Published:** 2025-05-09

**Authors:** Lamyia Anweigi, Iheb Ben Naceur, Jomana Awad, Mohamed Ahmeda, Noha Barhom, Faleh Tamimi

**Affiliations:** ^1^ College of Dental Medicine, QU Health Qatar University Doha Qatar; ^2^ Dentistry Newcastle University Newcastle upon Tyne UK

**Keywords:** artificial intelligence, hypodontia, natural language processing, patient outcomes, quality of life, tooth development

## Abstract

**Background:**

Natural language understanding (NLU), a subfield of artificial intelligence, focuses on the computational understanding of human language. This technology offers an objective and quantitative approach to analysing interviews in qualitative research. This study hypothesises that NLU can assess the impact of oral health on quality of life by analysing semi‐structured interviews.

**Objective:**

This study aimed to assess the utility of NLU in evaluating oral health‐related quality of life by analysing semi‐structured interviews with individuals diagnosed with hypodontia.

**Methods:**

A cross‐sectional qualitative study was conducted on 10 participants (aged 16–25 years) suffering from hypodontia. Semi‐structured interviews were transcribed and analysed using IBM Watson NLU text analysis. The analysis identified entities, keywords, sentiments (positive and negative) and emotions (joy, sadness, anger, fear and disgust) expressed in the interviews.

**Results:**

NLU analysis revealed a predominantly negative sentiment towards hypodontia and its management, with 93.2% of identified entities presenting a negative sentiment and only 6.8% showing a positive sentiment. Patient sentiment correlated inversely with age (*R* = −0.49), treatment waiting time (*R* = −0.22) and OHIP score (*R* = −20). Negative sentiments and sadness were most prominent when discussing the history of dental problems and feelings about their teeth, whereas joy and positive sentiments were expressed regarding successful dental work. Keywords associated with negative sentiment were primarily related to treatment length and delays.

**Conclusion:**

NLU effectively identified patients' negative sentiments and emotional responses to oral health conditions, demonstrating its potential as a valuable tool in qualitative dental research.

## Graphical abstract

Our study employs Natural Language Understanding (NLU) as a qualitative text analysis method, which is less biased, more time‐efficient, and resource‐saving than traditional methods. This study offers a data‐driven approach to understanding patients' sentiments and needs. These insights can guide clinicians in tailoring treatment plans, help policymakers improve dental care accessibility, and ultimately enhance patients' well‐being.

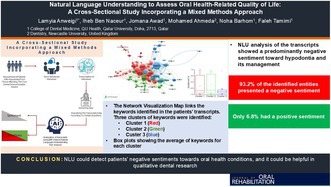

## 1 Introduction

Qualitative research methods in dental research are invaluable for understanding participants' perspectives in their own words. These methods generate unstructured textual data from semi‐structured or focus group interviews [1], which are typically recorded [2]. During analysis, a code is defined as a qualitative label (e.g., ‘staying healthy’ or ‘harmful’) used to identify and summarise the meaning of a text segment or theme relevant to the study aims or research questions [3, 4]. However, one major limitation of qualitative research is the subjective bias introduced by moderators and analysts when reporting results. An objective method to analyse transcripts could yield more valid and reliable findings.

Natural language processing (NLP), a subfield of artificial intelligence, automates qualitative text analysis by understanding and manipulating natural language text [5, 6]. NLP enables the analysis of significantly larger text databases compared to traditional methods. It has been applied to electronic health records [7, 8], medical literature [8], social media [9] and text messages [10]. Studies show that NLP can quickly and accurately identify key themes comparable to those derived from traditional qualitative analysis [6, 11].

In dental research, NLP has demonstrated promising results [12, 13, 14, 15]. It has enhanced craniofacial and dental phenotyping vocabularies using external phenotype terms from clinical records [14, 16, 17]. NLP has also been applied to analyse online forums of dental practitioners and patient reviews of dental treatments [16].

Despite its potential, few researchers have applied NLP to analyse semi‐structured interview transcripts in qualitative research [18, 19, 20]. In dentistry, no study to date has used NLP for this purpose.

Hypodontia, the congenital absence of fewer than six teeth, affects 2.3%–11.3% of the population [21]. It significantly impacts patients' quality of life (QoL), particularly their social and emotional well‐being [21, 22]. Understanding the effect of hypodontia on patients' QoL provides valuable insight into how the condition affects their daily lives and helps improve oral health care for these patients.

This study assessed the use of NLP in qualitative oral health research on hypodontia. It explored how NLP could evaluate patient QoL by identifying sentiment and emotion in semi‐structured interview transcripts, comparing these findings to qualitative analysis performed by experienced researchers. The null hypothesis was that natural language understanding (NLU) could not effectively detect patient sentiment or emotion related to oral health conditions compared to traditional qualitative methods.

## 2 Methodology

This cross‐sectional (incorporating mixed methods approach) research study was designed to assess the impact of hypodontia on quality of life using NLP. Ethical approval was obtained from the Cork University Hospital Ethical Committee (2007–2010, ECM5 (9)).

A group of patients with hypodontia was recruited from Cork Dental School and Hospital (Ireland). This teaching centre receives referrals for secondary‐ and tertiary‐level dental care from a population of approximately 1 million. The inclusion criteria were male and female patients at various stages of treatment with either mild, moderate or severe hypodontia diagnosed in primary school through the dental screening programme for children between the ages of 7 and 10 years old (Table S1). The most commonly used classification defines mild hypodontia as the congenital absence of 1–2 teeth, moderate hypodontia as the congenital absence of 3–5 teeth and severe hypodontia as the congenital absence of 6 or more teeth [23, 24].

The sample size for this study was not independently determined. However, it was based on the data set utilised in Meaney et al. [25]. In the original study, 10 participants were included, as this number was sufficient to reach data saturation; at this point, no new themes emerged from the interviews, per the standard qualitative research practices [26].

The same data set of 10 interview transcripts was reanalysed for the current study using natural language understanding (NLU) techniques. This approach enables a direct comparison between the insights derived from traditional qualitative methods and those generated through NLU, ensuring methodological consistency and evaluating NLU's effectiveness in qualitative dental research. Participants provided written consent to participate in the study (parental consent was obtained for participants under 18 years). The participants were informed that participation was voluntary. Subjects were assigned a unique study ID on all reporting to ensure confidentiality and anonymity.

Participants completed the 49‐item Oral Health Impact Profile (OHIP) questionnaire. The scores for this questionnaire range from 0 to 196, where a high score means a high impairment of OHRQoL [27].

Patients were then interviewed using a topic guide (Table S2) based on patient QoL themes, updated after each interview until saturation was reached. The semi‐structured interviews were all conducted by a single interviewer, digitally recorded and professionally transcribed.

We have followed the STROBE (Strengthening the Reporting of Observational Studies in Epidemiology) guidelines to conduct and report our research as a human observational study.

### 2.1 NLU Analysis of the Interview Transcripts

This study used the Watson Natural Language Understanding (WNLU) open‐access tool to analyse patient interviews. IBM Watson's natural language understanding was selected based on its excellent performance in intent classification, confidence scores, entity extraction and its ability to work on long sentences [28].

WNLU analysis of the transcribed interviews and the specific responses to topic guide questions provided an output that included the identification of entities and keywords in the text and the emotion and sentiment towards the interview, the specific entities and keywords and the specific questions. The sentiment (positive and negative) was represented as a continuous number ranging between −1.0 and 1.0, indicating participant disagreement or agreement with the context of the conversation. Five types of emotion were measured (sadness, joy, fear, disgust and anger) as continuous values ranging from 0.0 to 1.0 in proportion to the strength of each dimension of emotion (Figure S1).

### 2.2 Theme Identification Using Artificial Intelligence

To identify the main themes in the interview, we used the VOSviewer software (VOSviewer version 1.6.20) developed by Leiden University's Centre for Science and Technology Studies to identify the clusters of keywords in the transcripts of the whole interview. The mapping was set to binary counting and a minimum occurrence of a term of 1 to include all extracted terms.

The identified terms were added to the keywords provided by WNLU. The network visualisation occurs by linking the terms as in the interview transcript, using 10.00 visualisation and a minimum cluster size of 80. The keywords with 60 or more frequencies were excluded to create a more representative scheme.

**FIGURE 1:**
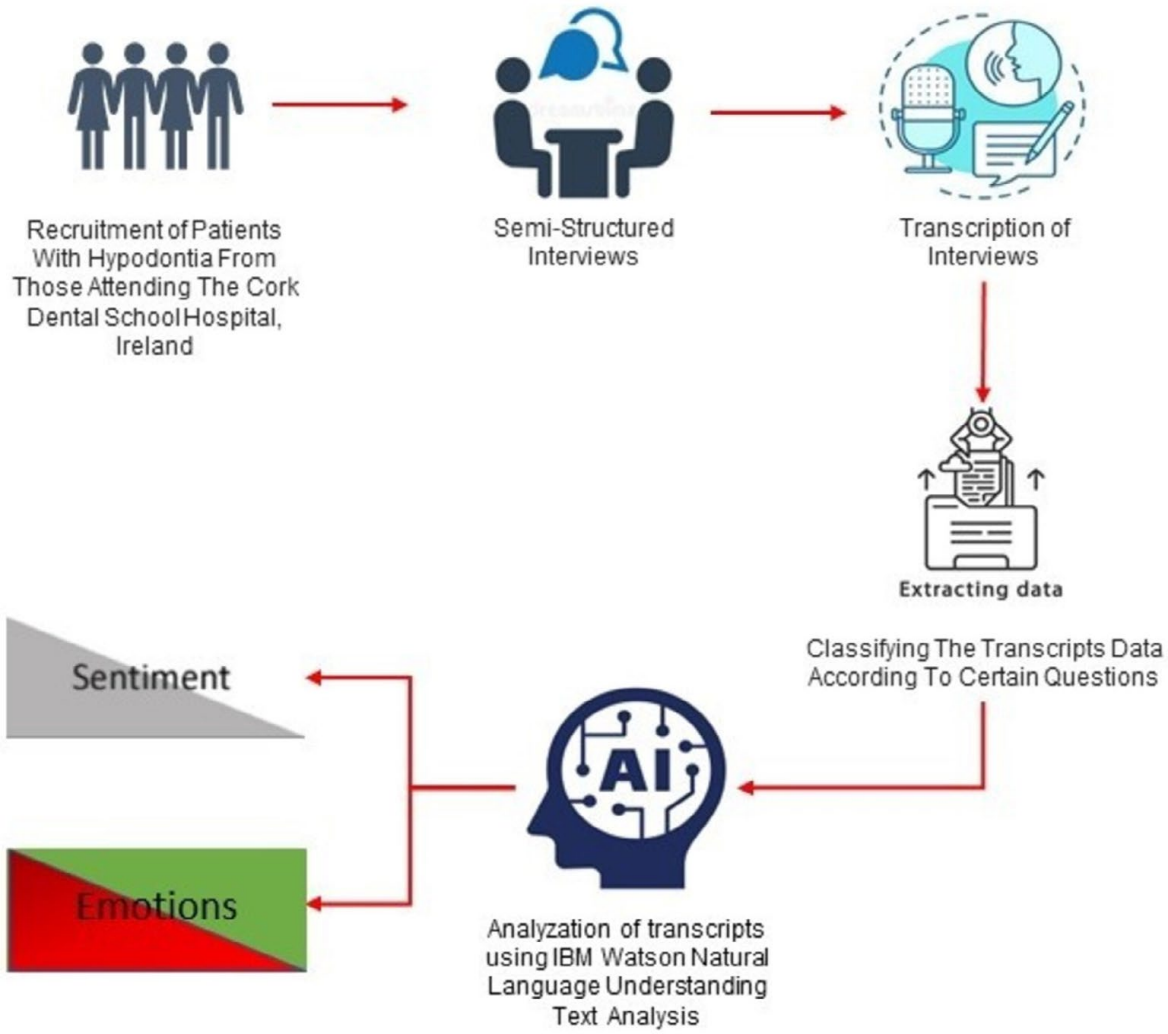
Flow chart of qualitative analysis to interview transcript using natural language understanding (NLU).

The results of the NLU analysis were compared with the traditional qualitative assessment of the transcripts performed by an independent group of researchers blinded to the NLU analysis (Figure 1).

### 2.3 Statistical Analysis

Pearson's correlation analysis was employed to assess the relationship between continuous variables. Friedman's test compared emotions and sentiments across questions and word clusters. All statistical analyses were performed using SPSS (version 22), with a significance threshold set at *p* < 0.05.

## 3 Result

All participants were Irish and were undergoing treatment, which varied according to hypodontia severity. The participants ranged from 16 to 25 years of age, five male and five female. The number of missing teeth ranged from 4 to 13. The participants' OHIP scores ranged between 24 and 143, and the delay in treatment was between 8 and 14 years (Table S3).

**FIGURE 2:**
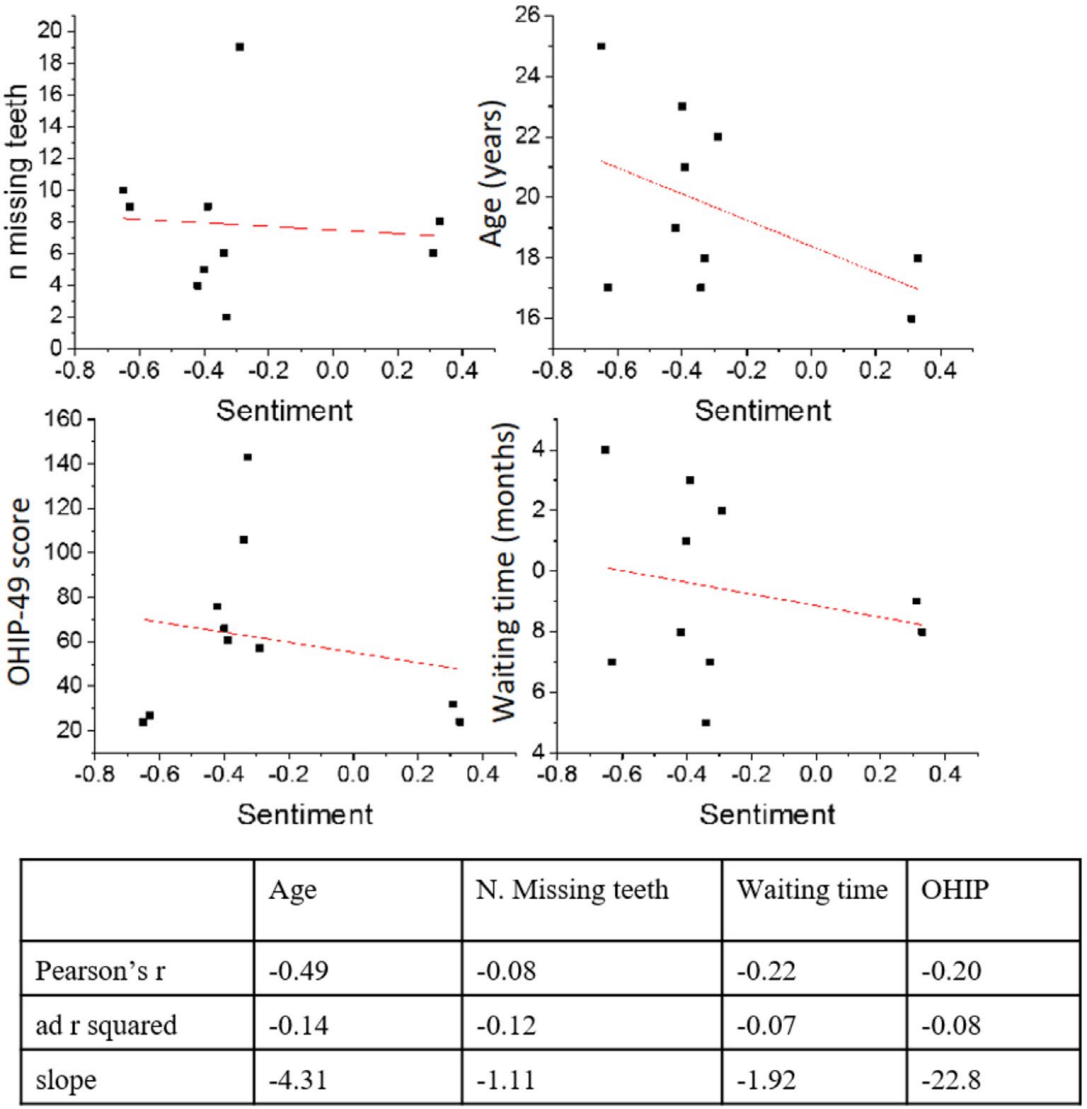
Correlation between the Sentiment Score on the one hand and the number of missing teeth, age, OHIP‐49 score, and waiting time on the other hand.

NLU analysis shows that out of the 10 participants, two presented an overall positive sentiment in the interview, and eight had an overall negative sentiment. The patient's overall sentiment was negatively correlated with the patient's OHIP score, age, number of missing teeth and treatment waiting time. This correlation was strong for patient age (*r* = − 0.49) but weaker for OHIP score (*r* = − 0.20) and treatment waiting time (*r* = −0.22) and very weak for the number of missing teeth (*r* = −0.08) (Figure 2).

**FIGURE 3:**
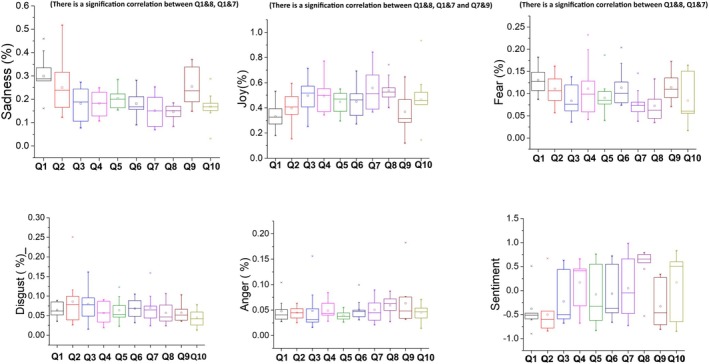
Box plots depicting the score for each emotional category (sadness, joy, fear, disgust and anger) as a function of each question as well as the average sentiment for each question for each patient.

The average sentiment for each interview question shows that four questions presented a mean of positive sentiment, whereas the other six had a mean of negative sentiment (Figure 3 and Figure S2). Questions that were answered with a negative sentiment were those on the ‘History of Dental Problems’ (Q1), ‘Feelings about Teeth and Dental Work’ (Q2), ‘Management of Dental Work’ (Q3), ‘Management of Treatment’ (Q5), ‘Expectations of Dental Work’ (Q6) and ‘Dental work problems’ (Q9). Questions that were answered with a positive sentiment were those on the ‘Provision of Dental Work’ (Q4), ‘Meeting Expectations’ (Q7), ‘Successful Dental Work’ (Q8) and ‘Future Dental Work’ (Q10).

The correlation analysis between the sentiment score for each question (Q) (Table 1) shows a significant correlation between the sentiment scores of Q1 and Q5, respectively. We also found a very strong correlation between Q6 and Q9, respectively. Moreover, Question 10 sentiment was significantly correlated with the OHIP score (*r* = 0.669 *p* = 0.034); this meant that patients with a higher level of impairment expressed more positive sentiment when discussing the future dental work to be done.

**TABLE 1:** Pearson correlation of question sentiments and OHIP scores, with positive correlations highlighted in green and negative correlations highlighted in red.

	Q1	Q2	Q3	Q4	Q5	Q6	Q7	Q8	Q9	Q10	OHIP
Q1: History of Dental Problem	1										−0.134
Q2: Feelings about Teeth and Dental Work	−0.065	1									0.043
Q3: Management of Dental Work	0.195	−0.154	1								−0.588
Q4: Provision of Dental Work	−0.108	0.316	−0.066	1							0.473
Q5: Management of Treatment	0.685*	0.517	0.21	−0.032	1						−0.411
Q6: Expectations of Dental Work	0.243	0.293	−0.398	0.274	0.322	1					0.086
Q7: Meeting Expectations	−0.595	0.127	−0.215	−0.13	−0.392	0.334	1				−0.179
Q8: Successful Dental Work	0.165	−0.49	0.451	−0.266	−0.195	−0.172	−0.041	1			−0.139
Q9: Dental Work Problematic	0.337	0.5	−0.315	0.502	0.426	0.888**	0.131	−0.15	1		0.282
Q10: Future Dental Work	−0.194	0.239	−0.518	0.564	−0.347	0.119	0.192	−0.34	0.331	1	0.669*

* Correlation is significant at the 0.05 level (2‐tailed).

** Correlation is significant at the 0.01 level (2‐tailed).

According to emotion analysis, the predominant feelings of the participants answering the interview questions were joy and sadness, followed by fear, disgust and anger, with two exceptions: answers from Questions 9 and 10 showed stronger anger than disgust. Patients were saddest and most fearful answering Question 1 (history of dental work), most joyful answering Question 7 (meeting expectations), most disgusted answering Question 3 (management of dental work) and most angry answering Question 9 (dental work problematic) (Table S4 and Figure S2).

**FIGURE 4:**
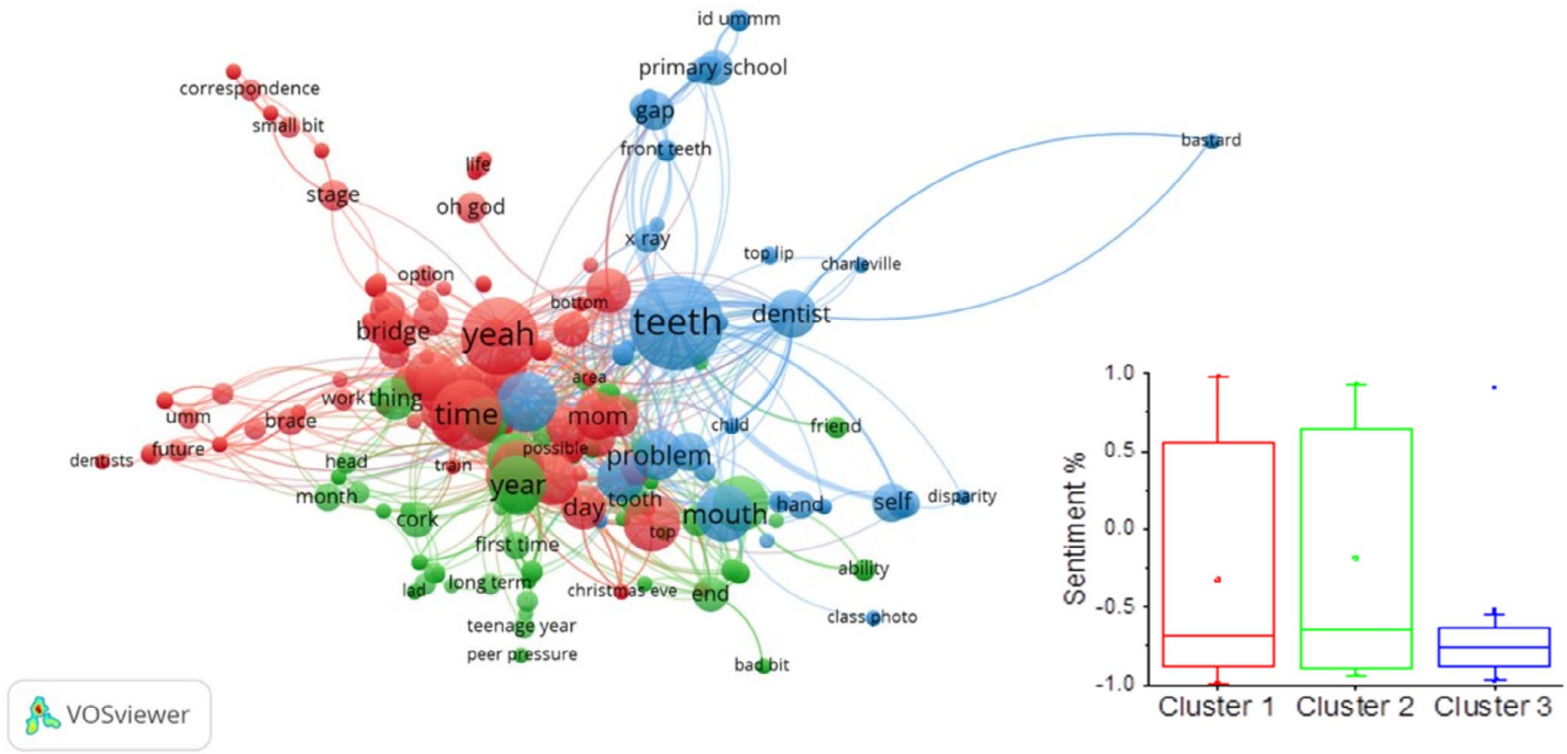
The network visualisation map links the keywords identified in the patients' transcripts. Three clusters of keywords were identified: Cluster 1 (red), cluster 2 (Green), cluster 3 (blue) and box plots showing the average of keywords for each cluster.

NLU analysis of the interview transcripts also identified specific keywords, their associated entities and sentiment. The relationship between these keywords was analysed by automatically clustering them into domains according to word–word relationships using the VOSviewer software (VOSviewer version 1.6.20). We found that keywords could be clustered into three main categories according to their relationships in the transcripts (Figure 4). A total of 369 terms were identified and clustered into three main themes.

### 3.1 Cluster 1 (Red Cluster)

This cluster included keywords related to clinical problems (e.g., biggest problem) such as pain, aesthetic dental concerns (e.g., ‘perfect smile’, ‘mouth’, ‘teeth’, ‘perfect teeth’), self‐awareness (e.g., ‘self‐conscious’, ‘notice’) and financial considerations (e.g., ‘finances’, ‘cost’, ‘money’) as well as treatment (e.g., ‘dental work’, ‘best treatment’, ‘dentist’, ‘decisions’, ‘bridges’) and treatment timeline (e.g., times, day, recent times, later stage, waiting room, hours, end of the day, long process, long wait) (Table S5).

### 3.2 Cluster 2 (Green Cluster)

The words identified in this cluster referred to the diagnosis of the condition (e.g., ‘first time’, ‘peg teeth’), including the site in which diagnosis took place (e.g., cork) as well as the treatment waiting time (‘dentist’, ‘waiting list’, ‘appointment’, ‘a lot of the dental appointments’, ‘loads of orthodontist appointments’, ‘a lot of the dental appointments’, ‘a good chunk of school’). The words identified in this cluster reflect the patient's frustration about their condition (e.g., ‘ash’, ‘Jesus mum’, ‘self‐image’) (Table S5).

### 3.3 Cluster 3 (Blue Cluster)

Keywords in this cluster referred mainly to the process of treatment (e.g., year, month, X‐ray, check‐ups, end product, bureaucracy, red tape), including the location in which treatment took place (e.g., Wicklow, Loughlinstown Hospital) (Table S5).

Sentiment analysis for the keywords identified in each cluster revealed significant negative sentiment for the keywords in Cluster 3 concerning appearance, treatment waiting time and dissatisfaction with the length of their treatment (Table S6).

## 4 Discussion

This study introduced the use of NLU to assess the impact of oral health conditions (i.e., hypodontia) on patients' quality of life. NLU analysis indicated a predominantly negative sentiment towards hypodontia and its management. The negative sentiment obtained by NLU analysis was correlated with a higher OHIP score, age and missing teeth. This could be explained by the fact that similar concerns (aesthetics, pain and finance), diagnosis, treatment waiting time and treatment process were expressed on the OHIP questionnaire regarding the negative impacts of hypodontia.

The correlation between older age and a more negative sentiment may be attributed to the patients' increased self‐awareness or self‐consciousness of their situation with age. This is consistent with previous reports on the adverse psychosocial effects of hypodontia. As they age, children become more aware and dissatisfied with their dental aesthetics and start feeling uncomfortable in social settings [25]. This also agrees with a previous study that observed a positive correlation between age, functional limitation and physical disability scores in patients with hypodontia [29].

The correlation between more missing teeth and a more negative sentiment may be related to aesthetics, speech and mastication abilities being more likely compromised with more missing teeth. As for the length of treatment waiting time, patients expect an immediate solution for their missing teeth as they are concerned that their gap will become more significant and more noticeable to others. Once patients realise the actual length of their treatment, they become more burdened, impacting their sentiments more negatively [30].

NLU analysis of the specific answers for each question shed light on patient frustrations with their dental conditions, treatments, the complications derived from the treatments, and the unmet expectations. History of Dental Problems (Q1) evoked a negative sentiment and the saddest and most fearful emotions, which could be related to the patients' self‐awareness about their situations and frustration about their condition. Feelings About Teeth and Dental Work (Q2), the Management of Dental Work (Q3), the Management of the Treatment (Q5) and Dental Work Problems (Q9) evoked negative sentiments. This could be explained by the patients' frustration about their current condition and unsuccessful dental work experiences. The Dental Work (Q6) expectations showed negative sentiment, which could relate to the unmet expectations of the patients. On the other hand, questions on the current provision of dental treatment (Q4), the met expectations (Q7), the success of the dental treatment (Q8) and future dental work (Q10) evoked joyful emotions and positive sentiments. This indicates that patients’ perceived anxiety and dissatisfaction might be overcome once treatments are successfully completed and their expectations are met.

The results of the OHIP questionnaire correlated significantly with the sentiment associated with the answers to questions on future dental work (Q10). This could indicate that patients with worse OHRQoL are more emotional about receiving future dental treatments, whereas those with better OHRQoL are less emotional about it.

An in‐depth text analysis of the keywords in the highlighted transcripts identified three main clusters addressing the following crucial issues concerning patients with hypodontia and oral health practice.

### 4.1 Patient Concerns (Aesthetics, Pain and Finance)

One of the keyword clusters identified in the interview was mainly linked to patients' concerns about aesthetics, pain and financial constraints. It has been well documented that a patient's perception of their appearance and deviation from the norm or minimum standard of acceptability [31, 32] is a significant factor in the decision to undergo dental treatment. The findings of this study mirror those of Davis et al. [31] and Meaney et al. [25], whereby patients indicated that cosmetic appearance was the most influential factor in the choice of current or future dental treatment [25, 31], whereby patients indicated that cosmetic appearance was the most influential factor in the choice of current or future dental treatment.

### 4.2 Diagnosis and Treatment Waiting Time

The second cluster of keywords identified in the interview was mainly related to the diagnosis of the condition and the waiting time for the treatment to start. Each participant was initially diagnosed in primary school during the routine dental screening. The benefits of such a program have been illustrated elsewhere [33]. Although context‐specific, this study demonstrates how important it is for patients with hypodontia to have access to early diagnosis and appropriate care promptly.

### 4.3 Treatment Process

Sentiment analysis of the keywords detected in cluster 3 revealed an overall negative sentiment of the participants towards the process of the treatment. Indeed, even though the initial diagnosis occurred in childhood, treatment did not commence until mid to late adolescence for all participants. This is in line with the previous qualitative analysis of the transcripts [25] that revealed the discontent of participants with the delays in treatment. The regions surveyed had no integrated or formalised service for managing patients with congenital teeth anomalies. Consequently, participants were transferred between waiting lists (e.g., from orthodontic treatment to restorative dental care), which resulted in the participants experiencing delays between initial diagnosis and treatment. Moreover, participants thoroughly described feeling forgotten during this period.

### 4.4 Comparison With Qualitative Research Methods

The natural language understanding (NLU) analysis in this study agrees with traditional qualitative research methods of analysis of the same transcripts. Traditional qualitative research methods also showed participants' dissatisfaction with treatment waiting time and concerns regarding appearance, understanding, condition and treatment process [25] (Table S7). The similarity in the findings between the traditional qualitative research and NLU indicated that NLU could be used in qualitative research in dentistry. This finding agrees with the study by Crowston et al. [11].

Patients with hypodontia have several treatment needs, as the symptoms of their condition can vary considerably. In most cases, they will need both orthodontic and restorative treatment. Recent trends illustrate that patients are becoming more aware of treatments, and expectations have risen dramatically. Patients are more involved in decision‐making as their rights come to the forefront of dental practice [34].

All patients should have access to an informed healthcare professional, and these providers work closely with patients. A combined clinic would allow for increased communication and cooperation among practitioners and patients and perhaps ease some of the anxiety and frustration illustrated by this group [22, 35].

The predominantly negative sentiment identified by WNLU towards hypodontia and the treatment process length is in agreement with previous studies using traditional methods [25, 30, 36, 37] that reported how the presence of hypodontia and the complexity of its treatment impacted negatively patients' quality of life.

Some limitations are essential to highlight. The current sample size, although reaching saturation, is relatively small and may limit the applicability of the findings to a broader population. Therefore, caution should be exercised when interpreting the results concerning individuals with hypodontia outside our study setting. A larger sample size would enhance the generalisability and allow for more robust conclusions to be drawn.

Additionally, the available patients willing to be treated were aged 16–25, further limiting the generalisability of the results to other age groups. Future research should aim to include a broader range of age groups. This would ensure a more representative sample and enable researchers to draw conclusions that can be applied to a broader population.

Another limitation is that WNLU does not comprehensively explain how it arrives at its results. Although the available resources support WNLU's reliability and validity [28], we still need a tool that explains how it reached each specific result.

## 5 Conclusion

NLU analysis can detect patients' negative sentiments towards an oral health condition such as hypodontia. This novel method could be helpful for qualitative research in dentistry.

## Author Contributions

Lamyia Anweigi: contributed to conception, design, data acquisition and interpretation; drafted and critically revised the manuscript and submitting process. Iheb Ben Naceur: CONTRIBUTED to conception and design, ran the sentiment analysis of the patients' interviews using IBM Watson NLU Text Analysis and revised the manuscript. Jomana Awad: contributed to conception and design, ran the sentiment analysis of the patients' interviews using IBM Watson NLU Text Analysis and revised the manuscript. Mohamed Ahmeda: contributed to conception and design, ran the sentiment analysis of the patients' interviews using IBM Watson NLU Text Analysis and revised the manuscript. Noha Barhom: data acquisition and interpretation, performed all statistical analyses and drafted. Faleh Tamimi: contributed to conception, data analysis, design and critically revised the manuscript. All authors gave final approval and agreed to be accountable for all aspects of the work.

## Conflicts of Interest

The authors declare no conflicts of interest.

## Supporting information


Appendix S1

## Data Availability

The data that support the findings of this study are available upon request from the corresponding author. The data are not publicly available due to privacy or ethical restrictions.
