# Supplementary material for: Natural Language Understanding to Assess Oral Health‐Related Quality of Life: A Cross‐Sectional Study Incorporating a Mixed Methods Approach
Source: J Oral Rehabil. 2025 May 9;52(9):1377–85. doi: 10.1111/joor.13986 (PMC12408959; doi:10.1111/joor.13986)
Supplement: Supplementary file 1 — Appendix S1 [file JOOR-52-1377-s001.docx]

**Natural language understanding to assess Oral Health-Related Quality of life**

Lamyia Anweigi, Iheb Ben Naceur, Jomana Awad, Mohamed Ahmeda, Noha Barhom, Faleh Tamimi.

**Appendix tables and figures:**

**Appendix Table 1.** The characteristics of the participants.

| **Patient Id** | **Gender** | **Age** | **OHIP summary score** | **N Missing Teeth** | **Delay in treatment** |
| --- | --- | --- | --- | --- | --- |
| 1 | Male | 16 | 32 | 6 | 9 |
| 2 | Male | 25 | 24 | 10 | 14 |
| 3 | Male | 18 | 24 | 8 | 8 |
| 4 | Male | 23 | 66 | 5 | 11 |
| 5 | Male | 21 | 61 | 9 | 13 |
| 6 | Female | 17 | 27 | 9 | 7 |
| 7 | Female | 19 | 76 | 4 | 8 |
| 8 | Female | 17 | 106 | 6 | 5 |
| 9 | Female | 18 | 143 | 2 | 7 |
| 10 | Female | 22 | 57 | 19 | 12 |

**Appendix Table 2.** The interview topic guide.

| **Topic** | **Aspects to be explored** |
| --- | --- |
| Name & D.O.B. |  |
| 1. History of Dental Problem | When did it start?  Who noticed? How? |
| 1. Feelings about Teeth and Dental Work 2. Management of Dental Work | At that time? Start to now!  Missing teeth/appearance 1^st^ preference?  Other options / preferences?  Choices available  Family history an influence |
| 1. Provision of Dental Work | Source of idea of getting dental work done?  (Self, parents, significant others, dentist, other health professional?)  What factors in decision to get Dental work done?  (Info available; finance – self / govt; sense of self – personal / professional; family; dentist; disease, peers)  Any Obstacles? – Waiting lists |
| 1. Management of Treatment | Did you have enough knowledge information / understanding?  What do you think of the whole process  Has that changed over the years Childhood to present?  What could have helped re above if any shortcomings? |
| 1. Expectations of Dental Work | What will improve in your situation: Physical (speech, chewing) / Psychological affect as it improves: Appearance / Social /  Professional / impact on other aspects of health?  Any changes over the years |
| 7. Meeting Expectations | Met – explore above aspects  Unmet – explore above aspects  Any difference between different types of treatment – different dentists  Explore access / adequacy / satisfaction  Suggestions for improvements?  Self – care: how did you look after RPD / mouth etc |
| 8. Successful dental work successful | - care - access to professional support i.e. dentist, orthodontist, dental technician  - access to financial support (from parents to own)  - any others |
| 9. Dental work problematic | Explore problems identified – consider factors  listed above  Time consuming work  Painful procedures |
| 10. Future Dental Work | What are your expectations into the future:  - Appearance (influence of peers)  - Social comfort with peers etc.  - Care  - Access to professional support i.e. dentist, orthodontist, restorative etc. dental technician  - Access to financial support (from parents to own)  - Any others |

***
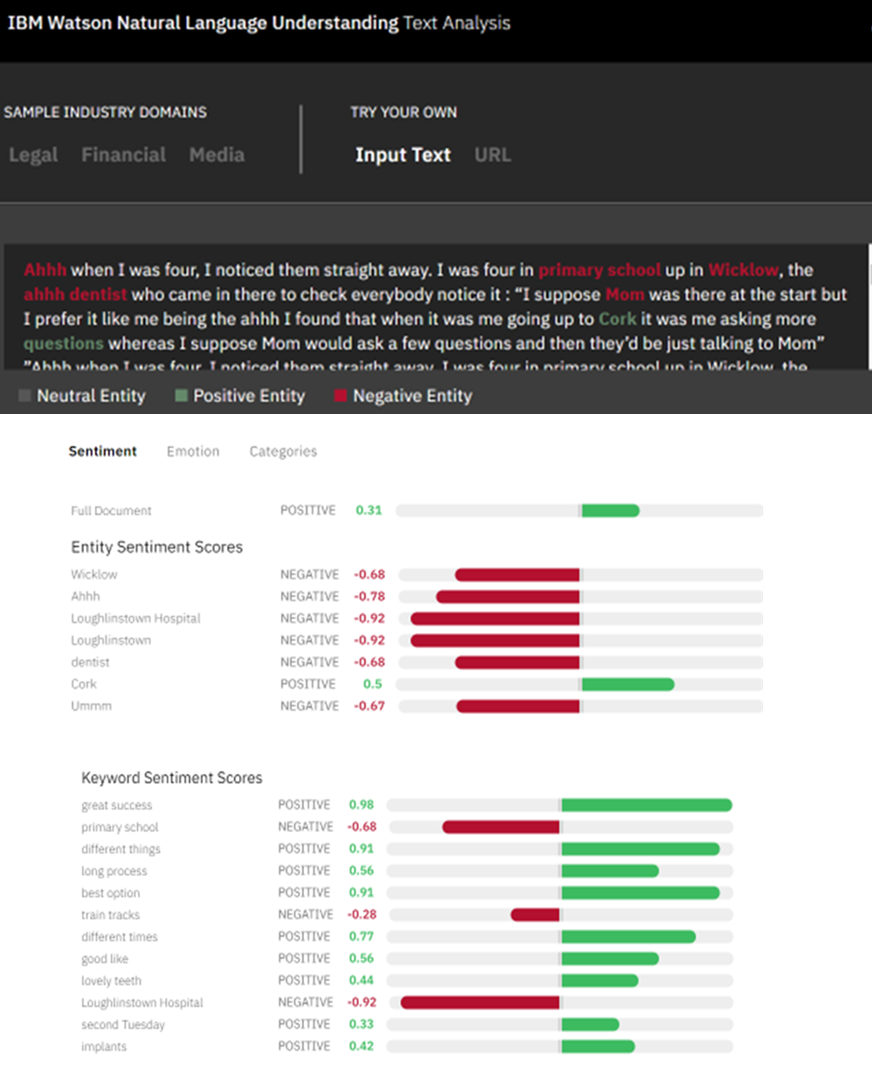
***

**Appendix Figure 1*.*** IBM WNLU detection of keywords and entities (above), and sentiment analysis of keywords and entities

**Appendix Table 3.** Characteristics, OHIP score and the NLU sentiment score for each patient.

| Patient# | Gender | Age | OHIP Score | Number of Missing Teeth | Delay in treatment (years) | Sentiment score |
| --- | --- | --- | --- | --- | --- | --- |
| 1 | Male | 16 | 32 | 6 | 9 | 0.31 |
| 2 | Male | 25 | 24 | 10 | 14 | -0.65 |
| 3 | Male | 18 | 24 | 8 | 8 | 0.33 |
| 4 | Male | 23 | 66 | 5 | 11 | -0.4 |
| 5 | Male | 21 | 61 | 9 | 13 | -0.39 |
| 6 | Female | 17 | 27 | 9 | 7 | -0.63 |
| 7 | Female | 19 | 76 | 4 | 8 | -0.42 |
| 8 | Female | 17 | 106 | 6 | 5 | -0.34 |
| 9 | Female | 18 | 143 | 2 | 7 | -0.33 |
| 10 | Female | 22 | 57 | 19 | 12 | -0.29 |
| OHIP Score: The Oral Health Impact Profile score ranges from 0 to 196, and higher scores indicate greater impairment of oral health-related quality of life. | | | | | | |


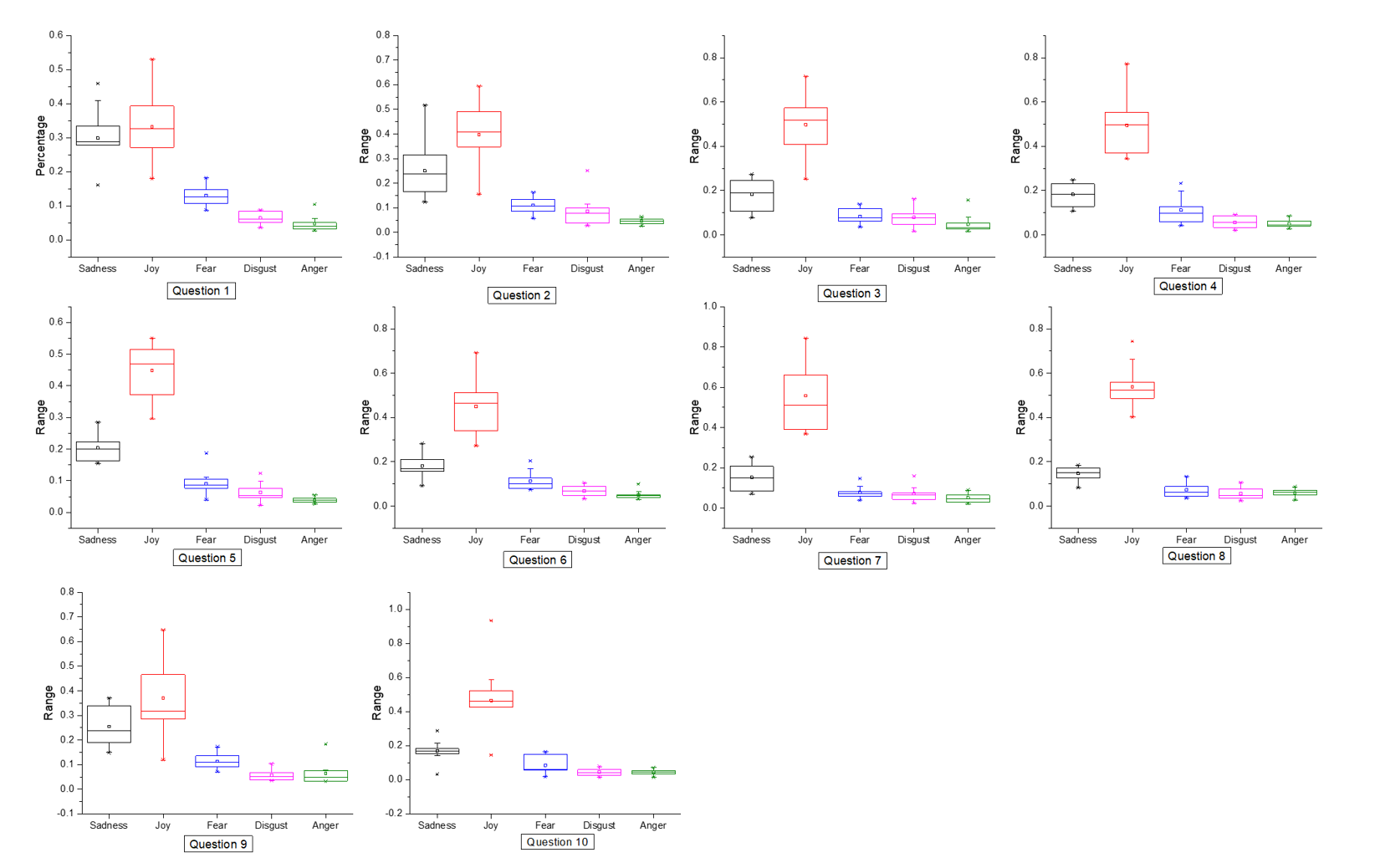


**Appendix Figure 2.** Emotional composition of response to Questions 1-10.

**Appendix Table 4.** Distribution of emotions (sadness, joy, fear, disgust, anger) in response to each question.

| Question | Emotion | Sadness | Joy | Fear | Disgust | Anger |
| --- | --- | --- | --- | --- | --- | --- |
| Q1 | AVG | 29.89% | 33.14% | 13.05% | 6.43% | 4.77% |
|  | SD | 8.43% | 9.53% | 3.07% | 1.83% | 2.16% |
| Q2 | AVG | 25.01% | 39.67% | 11.08% | 8.59% | 4.50% |
|  | SD | 11.24% | 12.74% | 3.23% | 6.23% | 1.16% |
| Q3 | AVG | 18.23% | 49.80% | 8.38% | 7.95% | 4.83% |
|  | SD | 6.40% | 14.86% | 3.12% | 4.17% | 3.98% |
| Q4 | AVG | 18.24% | 49.47% | 11.12% | 5.64% | 4.89% |
|  | SD | 5.00% | 11.91% | 5.96% | 2.61% | 1.65% |
| Q5 | AVG | 20.36% | 44.87% | 9.05% | 6.39% | 3.91% |
|  | SD | 3.86% | 8.04% | 3.82% | 2.77% | 0.92% |
| Q6 | AVG | 18.13% | 45.00% | 11.36% | 6.81% | 5.05% |
|  | SD | 4.91% | 12.12% | 4.04% | 2.47% | 1.86% |
| Q7 | AVG | 15.20% | 55.80% | 7.71% | 7.02% | 5.08% |
|  | SD | 6.08% | 15.96% | 2.97% | 3.56% | 2.27% |
| Q8 | AVG | 14.66% | 53.73% | 7.26% | 5.69% | 5.93% |
|  | SD | 2.88% | 9.47% | 3.27% | 2.82% | 1.70% |
| Q9 | AVG | 25.48% | 36.94% | 11.39% | 5.76% | 6.33% |
|  | SD | 7.69% | 14.08% | 3.08% | 2.20% | 4.33% |
| Q10 | AVG | 16.89% | 46.54% | 8.44% | 4.51% | 4.52% |
|  | SD | 6.03% | 20.20% | 4.92% | 2.08% | 1.46% |
| Q1-History of Dental Problem      Q2-Feelings about Teeth and Dental ‎Work    Q3-Management of Dental Work  Q4-Provision of Dental Work      Q5-Management of Treatment    Q6-Expectations of Dental Work  Q7-Meeting Expectations             Q8- Successful   Dental Work          Q9-Dental work problematic  Q10-Future Dental Work | | | | | | |

**AVG:** Average, **SD:** Standard Deviation

**Appendix Table 5.** Summary of the linked Keyword identified for each cluster using VOSviewer for interview transcripts.

| Keyword | Average | Cluster 1  Red | Keyword | Average | Cluster 2  Green | Keyword | Average | Cluster 3  Blue |
| --- | --- | --- | --- | --- | --- | --- | --- | --- |
| ahhh | 0.91 | 1 | Ahhh things | 0.7 | 2 | ahhh dentist | -0.68 | 3 |
| appointment | -0.685 | 1 | bad bits | 0.79 | 2 | big smile | 0.73 | 3 |
| area | -0.57 | 1 | best option | 0.91 | 2 | boarding school ummm | -0.9 | 3 |
| best treatment | 0.93 | 1 | bit | -0.72 | 2 | bottom lip | -0.88 | 3 |
| bridge | -0.68 | 1 | bit of a minefield | -0.83 | 2 | Charleville | -0.89 | 3 |
| bridges | 0.68 | 1 | chunk of time | -0.81 | 2 | check-ups | -0.88 | 3 |
| Christmas Eve | -0.51 | 1 | condition | -0.69 | 2 | conscious teen | -0.91 | 3 |
| cost | -0.72 | 1 | Cork | 0.545 | 2 | dentist | -0.77 | 3 |
| course | 0.98 | 1 | couple of people | -0.89 | 2 | different things | 0.91 | 3 |
| day | -0.54 | 1 | dentist x | -0.94 | 2 | different times | 0.77 | 3 |
| decisions | 0.87 | 1 | family | -0.89 | 2 | fillings | -0.88 | 3 |
| dental work | -0.68 | 1 | first time | 0.59 | 2 | gap | -0.88 | 3 |
| end of the day | -0.795 | 1 | good chuck of school | -0.64 | 2 | gravity of the situation of what missing teeth | -0.74 | 3 |
| end product | 0.77 | 1 | happy like | -0.84 | 2 | kind of public speaking | -0.55 | 3 |
| euro return kind | -0.79 | 1 | Jesus ummm | -0.9 | 2 | kind of self-consciousness | 0.89 | 3 |
| finances | -0.9 | 1 | kind of a lot of people | 0.85 | 2 | know | -0.7 | 3 |
| good Christmas present | 0.83 | 1 | loads of orthodontist appointments | -0.92 | 2 | long-time kind | 0.62 | 3 |
| great success | 0.98 | 1 | long-term | 0.67 | 2 | Loughlinstown Hospital | -0.92 | 3 |
| half years | -0.56 | 1 | lot of the dental appointments | -0.6 | 2 | Mam | -0.89 | 3 |
| hour | -0.8 | 1 | nobody | -0.9 | 2 | mother | -0.83 | 3 |
| hours | -0.8 | 1 | peer pressure | -0.85 | 2 | mouth | -0.88 | 3 |
| kind of an arrangement | -0.57 | 1 | peg | -0.94 | 2 | much notice | 0.7 | 3 |
| kind of money | -0.91 | 1 | perfect like | 0.93 | 2 | normal stuff | -0.88 | 3 |
| last year | 0.66 | 1 | perfect smile | -0.83 | 2 | orthodontist appointments | -0.97 | 3 |
| later stage | -0.89 | 1 | permanent teeth | 0.64 | 2 | Part of the problem | -0.64 | 3 |
| less hassle | -0.69 | 1 | proper treatment | 0.52 | 2 | primary school | -0.68 | 3 |
| long process | 0.56 | 1 | self-image | -0.65 | 2 | Primary School | -0.63 | 3 |
| long wait | -0.69 | 1 | tooth | -0.94 | 2 | problems | 0.91 | 3 |
| lot of baby teeth | -0.88 | 1 | Transition Year | 0.52 | 2 | regular check-ups | -0.64 | 3 |
| lot of my teeth | -0.89 | 1 | year top | 0.78 | 2 | school child | -0.64 | 3 |
| mom | 0.88 | 1 | young girl | 0.59 | 2 | school whne | -0.84 | 3 |
| negative experience of bureaucracy | -0.93 | 1 |  |  |  | self-consciousness | -0.73 | 3 |
| normal size teeth | -0.92 | 1 |  |  |  | sixth class | -0.9 | 3 |
| obscure process | -0.94 | 1 |  |  |  | teeth | -0.7933 | 3 |
| pain | -0.9 | 1 |  |  |  | teeth Ummm | -0.55 | 3 |
| people | 0.5 | 1 |  |  |  | top lip | -0.54 | 3 |
| problem | -0.86 | 1 |  |  |  | Tralee | -0.9 | 3 |
| questions | 0.5 | 1 |  |  |  | ummm ahhhh | -0.67 | 3 |
| real life | -0.61 | 1 |  |  |  | Wicklow | -0.68 | 3 |
| recent times | 0.73 | 1 |  |  |  | x-ray | -0.88 | 3 |
| red tape pisses | -0.99 | 1 |  |  |  | x-rays | -0.88 | 3 |
| school | -0.88 | 1 |  |  |  | problem | -0.86 | 3 |
| Secondary School | -0.92 | 1 |  |  |  |  |  |  |
| short trip | -0.73 | 1 |  |  |  |  |  |  |
| small bit | -0.9 | 1 |  |  |  |  |  |  |
| small bit of difficulty | -0.92 | 1 |  |  |  |  |  |  |
| stage | 0.93 | 1 |  |  |  |  |  |  |
| stuff | -0.88 | 1 |  |  |  |  |  |  |
| times | 0.56 | 1 |  |  |  |  |  |  |
| treatment | -0.66 | 1 |  |  |  |  |  |  |
| Umm | -0.77 | 1 |  |  |  |  |  |  |
| ummm | -0.71 | 1 |  |  |  |  |  |  |
| waiting list | -0.66 | 1 |  |  |  |  |  |  |
| waiting room | -0.8 | 1 |  |  |  |  |  |  |
| way schools | -0.57 | 1 |  |  |  |  |  |  |

**Appendix Table 6.** The frequency of sentiment each cluster/theme using IBM KEYWORD

|  |  | 1 Frequency | |
| --- | --- | --- | --- |
|  | Count of key-word /theme | Negative | Positive |
| 1 | Importance of aesthetics | 34 (67%) | 17 (33%) |
| 2 | Waiting time for treatment | 35 (74%) | 12 (26%) |
| 3 | Transition from childhood to adulthood | 30 (79 %) | 8 (21%) |

**Appendix Table 7.** Comparison between the Natural Language Understanding (NLU) and Qualitative Text Analysis

| Natural Language Understanding (NLU) analysts of the transcripts | **Key findings of** Qualitative Text Analysis using NVivo (Meaney et al. 2012) |
| --- | --- |
| Predominantly negative sentiment in patients with hypodontia (average sentiment score of patients with hypodontia was negative (-0.37275).  There is a predominantly negative sentiment towards hypodontia and its management; 93.2 % of the entities identified by the NLU in the transcript were related to a negative sentiment, while only 6.8 % had a positive sentiment. | Hypodontia has a negative impact on a patient's quality of life |
| keywords clustered under the category related to aesthetics were mainly associated with negative sentiment (-0.31926). | Hypodontia patients have concerns about their appearance. |
| keywords clustered under the category related to treatment time were mainly associated with negative sentiment (-0.5198). | Hypodontia patients in the study presented dissatisfaction with treatment waiting time |
| keywords clustered under the category related to the transition to adulthood were mainly associated with negative sentiment (-0.24943). | The role of hypodontia patients in the treatment decision-making process changes signiﬁcantly as they move from childhood to adulthood. |
